# Masticatory muscle changes on magnetic resonance imaging of dogs with *Neospora caninum* compared to meningoencephalitis of unknown origin

**DOI:** 10.3389/fvets.2024.1517256

**Published:** 2025-01-07

**Authors:** Jessica Zilli, Kathryn Fleming, Chloe Fisher, Tim Sparks, Tom Harcourt-Brown, Edward Ives

**Affiliations:** ^1^Anderson Moores Veterinary Specialists, Linnaeus Veterinary Limited, Winchester, United Kingdom; ^2^Langford Vets, Small Animal Referral Hospital, Bristol, United Kingdom; ^3^Eastcott Veterinary Referrals, Swindon, United Kingdom; ^4^Waltham Petcare Science Institute, Waltham on the Wolds, Melton Mowbray, United Kingdom

**Keywords:** neosporosis, MUO, canine, MRI, myopathy

## Abstract

Infectious meningoencephalitides represent an important differential diagnosis for meningoencephalitis of unknown origin (MUO) in dogs. Treatment of the latter requires immunosuppression, but laboratory test results for infectious agents may take several days to return. This study investigated whether the presence of masticatory muscle changes on magnetic resonance imaging (MRI) of the head can be used to distinguish dogs with neosporosis from those with MUO at the time of diagnosis. Cases diagnosed with neosporosis or MUO at two referral centers in the United Kingdom (UK) were retrospectively collected. Clinical data were reviewed, and each MRI study was blindly assessed by a radiologist, a neurologist, and a neurology resident for the presence of masticatory muscle changes by consensus opinion. Statistical analysis was performed on obtained data. Twenty-two neosporosis cases and 23 MUO cases were enrolled. In the neosporosis group, six dogs (27%) had masticatory muscle changes, compared to one dog (4%) in the MUO group (*p* = 0.047). All six neosporosis cases had bilateral, multifocal, T2W and FLAIR hyperintense, contrast enhancing muscular changes, with three having concurrent masticatory muscle atrophy. The only MUO case with muscle changes had a mild, focal, unilateral temporal muscle lesion which was only visible in the T1W post-contrast images. Within the neosporosis group, dogs with masticatory muscle lesions had significantly higher cerebrospinal fluid WBC counts (*p* = 0.017) and protein concentrations (*p* = 0.025) compared to those without muscle changes. In conclusion, characteristic bilateral, multifocal masticatory muscle changes should raise the index of suspicion for neosporosis in dogs with an imaging diagnosis of meningoencephalitis and starting early antimicrobial treatment is recommended. However, the absence of masticatory muscle abnormalities does not exclude active *Neospora caninum* infection. In these cases, whether immunosuppressive or antimicrobial treatments are started prior to receiving further test results should still be based on the clinical status of the animal and index of suspicion using a combination of all available clinical information at that time.

## 1 Introduction

Inflammatory encephalopathies are a frequent cause of brain disease in dogs and can be divided into two broad categories: infectious aetiologies, and suspected immune-mediated aetiologies for which an infectious agent cannot be identified. In dogs, the most common inflammatory disorder of the central nervous system (CNS) is the group of diseases termed meningoencephalitis of unknown origin (MUO)/meningoencephalitis of unknown etiology (MUA) (1, 2). These are inflammatory, non-infectious diseases involving the brain and/or the spinal cord and their surrounding meninges. Their etiology is suspected to be immune-mediated and this is supported by a predominance of major histocompatibility complex class II and CD3 antigen-positive T-lymphocytes on histopathology and their response to immunosuppressive treatment (2, 3). Overall, within the MUO group, three predominant subtypes of encephalopathy are included based on breed predisposition, MRI findings and histopathologic characteristics: granulomatous meningoencephalitis (± meningomyelitis), necrotising meningoencephalitis and necrotising leucoencephalitis (1, 2, 4). However, it has been recently proposed that these subtypes may coexist or represent a continuum of the same disease process (5, 6).

Infectious meningoencephalitis is less common in dogs than MUO but represents an important differential diagnosis (7, 8). In particular, active infection by the protozoan *Neospora caninum* can result in intraparenchymal magnetic resonance imaging (MRI) changes that can mimic those seen in MUO (8–10). Although this form of protozoal meningoencephalitis only accounts for an estimated 2.25% of dogs diagnosed with meningoencephalitis in general (11), it still represents a risk for the overall dog population and particularly those in rural environments (12–14). Since definitive MUO diagnosis is obtained only by histopathology and brain biopsy remains infrequently performed in veterinary medicine despite more recent reports of its use (15–21), infectious aetiologies such as neosporosis should ideally be excluded before starting immunosuppressive treatment. This is to avoid promoting disease progression, with a recent study reporting a worse prognosis in dogs with neosporosis that were treated with prednisolone prior to diagnosis (8). To confirm the diagnosis of protozoal meningoencephalitis, serology and/or polymerase chain reaction (PCR) testing is required. However, the time required to run these tests and receive the results may potentially result in a delay in starting the most effective treatment for either MUO or *Neospora caninum.* In the authors’ experience, three to seven days are typically required to obtain serology results and three to five days are required for PCR testing. Considering that the prognosis for MUO remains poor to guarded, with up to one third of these dogs reported not to survive the first 72 h following diagnosis (1, 22, 23), early immunosuppressive treatment may be important to provide the best outcome (24). Therefore, looking for additional disease markers which may help in reaching an early diagnosis should be investigated.

*Neospora caninum* is a protozoal parasite which may cause meningoencephalomyelitis, polyradiculoneuritis and/or myopathy in the same host, even in the absence of clinical signs related to myositis (22). Therefore, cases with *Neospora* infection can present with skeletal muscle atrophy as a result of primary myositis and/or secondary to radiculoneuropathy and resultant denervation. A previous study investigated the use of serum muscle enzymes at the time of presentation to distinguish between MUO and *Neospora* infection and guide treatment decision making (25). In a similar manner, the presence of concurrent masticatory muscle changes in neosporosis cases may support clinical decision making at the time of imaging by helping to distinguish between neosporosis and MUO at an earlier stage than serologies and/or PCR results. Therefore, the aim of the current study was to assess the presence or absence of masticatory muscle changes in the MRI studies of dogs diagnosed with MUO compared to dogs with a final diagnosis of neosporosis. The hypothesis being that, unlike dogs with MUO, dogs with neosporosis may have concurrent masticatory muscle changes visible on MRI of their heads, which could allow differentiation between these diseases at the time of investigation and, in turn, early initiation of appropriate treatment.

## 2 Materials and methods

The clinical records of the Neurology and Neurosurgery service of Anderson Moores Veterinary Specialists (AMVS) and Langford Vets Small Animal Referral Hospital, between November 2015 and October 2023, were retrospectively reviewed, and two study groups, named “neosporosis” and “MUO,” were established. To be included in either group, the animals needed to have complete clinical history, neurological signs compatible with focal or multifocal brain disease, a brain MRI study that included the masticatory muscles (temporalis, masseter, pterygoids, ± digastricus) within the field of view, serologic ± PCR testing for *Neospora caninum*, and no clinical or imaging evidence of trigeminal nerve disease (i.e., dropped jaw, abnormal facial sensation). The administration of corticosteroids prior to investigations was also recorded for all dogs of each group.

To identify the cases to be included in the “neosporosis” group, the databases of both institutions were first searched for dogs that were tested for *Neospora caninum* using immunofluorescent antibody testing (IFAT) in serum and/or PCR in CSF. Those cases that had either a positive IFAT (at ≥1:800 dilution) and/or a positive PCR were only included in the “neosporosis” group if they also fulfilled the above-mentioned inclusion criteria and had a final diagnosis of *Neospora caninum* meningoencephalitis.

A comparable number of cases diagnosed with MUO was then retrieved from the AMVS database based on signalment (i.e., predisposed breeds, age > 6 months), history, clinical signs (consistent with focal or multifocal brain disease), brain MRI findings (i.e., single or multiple intra-axial hyperintense lesions on T2-weighted images), CSF analysis (presence of a pleocytosis with >50% mononuclear cells – monocytes/lymphocytes), in addition to a negative result of a *Neospora caninum* IFAT (at 1:50 dilution) ± PCR (1). Dogs for which CSF sampling was deemed contraindicated at the time of diagnosis due to clinical and/or imaging findings consistent with increased intracranial pressure (ICP), but that otherwise matched all other inclusion criteria for MUO (including a negative result of the *Neospora caninum* IFAT ± PCR), were still included. Dogs with incomplete clinical records (medical history or neurological examination), with incomplete brain MRI studies (see below), or absent *Neospora* testing were excluded from both groups. All the aforementioned clinical information was recorded in an Excel data spreadsheet, in addition to the breed, age, sex, neuter status, time from onset of signs to clinical presentation, neuroanatomic localization and serum muscle enzymes level (CK and AST) for each dog.

The MRI studies were performed with different 1.5 T MRI scanners over the years spanned by the data collection (Philips Intera and Philips Achieva at AMVS, and Philips Intera, Philips Symphony and Philips Ingenia at Langford Vets). Each MRI study had to include at least sagittal and transverse T2-weighted (T2W), transverse T2-weighted fluid attenuated inversion recovery (T2W FLAIR), transverse T1-weighted (T1W), and transverse and dorsal or 3D T1W post-contrast sequences. T2W dorsal sequences were also assessed if available. All identifying data were removed from the MRI studies, which were randomized and then assessed by a board-certified neurologist (E.I.), a board-certified radiologist (K.F.) and a neurology resident (J.Z.) who were blinded to the clinical information and diagnosis for each case during assessment. Given the natural variation in masticatory muscle conformation and bulk between different breeds, the authors were not blinded to breed during MRI assessment to allow for a more accurate assessment for masticatory muscle atrophy. For both groups, each MRI scan was assessed for the presence or absence of changes in masticatory muscle signal intensity, muscle volume and contrast enhancement pattern. The observers were asked only to evaluate the muscle changes and to disregard the appearance of the brain. Where present, the muscular changes were classified based on the localization as “focal” or “multifocal,” “unilateral or bilateral,” and based on their severity as absent (0), mild (1), or severe (2) depending on the signal intensity on T2W images (26). The presence or absence of subjective muscle atrophy or masticatory muscle asymmetry was assessed, and the individual muscles affected by any visible changes were recorded (i.e., temporalis, masseter, pterygoid and digastricus). The MRIs of all dogs were also assessed to ensure that there was no visible trigeminal nerve pathology. All the data were collected as a consensus, meaning that the three clinicians assessed all the MRIs together, the sequences were always positioned in the same orientation and order, using equivalent window levels during assessment, and all assessors had to agree on the presence of any potential abnormalities before they were considered relevant.

### 2.1 Statistical analysis

Data were collected in an Excel table and submitted for statistical analysis to the Waltham Petcare Science Institute. The neosporosis and MUO groups were compared to identify any differences in continuous/ordinal data (age, time from onset to presentation, CK activity, AST activity, CSF white blood cell (WBC) count, CSF protein concentration) using Mann–Whitney tests adjusted for ties. These data were summarized by medians and ranges. Dog age was also compared between absence or presence of masticatory muscle atrophy with a Mann–Whitney test adjusted for ties. Categorical data (sex, reported neuromuscular neurolocalization, *Neospora caninum* PCR result, severity of muscle changes, focal/multifocal changes, unilateral/bilateral changes, presence/absence of masticatory muscle atrophy, symmetry of the masticatory muscle bulk, contrast enhancement) were summarized as frequencies and percentages and compared between groups using Fisher Exact tests. Sensitivity, specificity and odds ratio for the presence of muscle changes were calculated. Significance was taken as *p* < 0.05. Analysis was conducted in Minitab21.

Within the neosporosis group, dogs with masticatory muscle changes (severity grades 1 and 2) were compared to dogs with no visible changes in masticatory muscle MRI signal intensity (severity grade 0) in order to assess for the presence of any statistically significant differences in age, time from onset to presentation, CK and AST activities, CSF WBC count, CSF protein concentration and PCR results.

## 3 Results

A total of 29 cases of neosporosis were initially extracted from the databases of both referral hospitals. Of these, seven dogs were excluded because of absent (*n* = 4) or incomplete (*n* = 3) MRI studies. Similarly, 29 dogs from an initial subset of 52 cases diagnosed with MUO were excluded from the MUO group because of insufficient MRI sequences available (*n* = 14), unremarkable CSF findings (*n* = 9), non-mononuclear/lymphocytic pleocytosis (*n* = 3), absent Neospora testing (*n* = 2) or positive results of the Neospora serology (*n* = 1). Overall, 22 dogs were included in the neosporosis group and 23 in the MUO group.

### 3.1 Signalment

In the neosporosis group, dogs of the following breeds were included: Greyhound (5/22; 23%), Labrador Retriever (3/22; 14%), Lurcher (2/22; 9%), French Bulldog (2/22; 9%), and one dog for each of the following: Border Collie, Boxer, Cavalier King Charles Spaniel, Cavapoo, Cocker Spaniel, Crossbreed, Doberman Pinscher, Golden Retriever, Italian Mastiff and Old English Sheepdog. Nineteen dogs (86%) were male (13 neutered and six entire), and three dogs (14%) were female (all neutered) (Supplementary Table S1). The median age at presentation was six years old (range: 0.33–12 years).

In the MUO group, 16 breeds were represented: Cockapoo (4/23; 17%), Chihuahua (2/23; 9%), Maltese (2/23; 9%), Pomeranian (2/23; 9%), Pug (2/23; 9%), and one dog for each of the following: Biewer Terrier, Border Collie, Cavapoo, Cocker Spaniel, Crossbreed, Japanese Chin, Labrador Retriever, Miniature Dachshund, Miniature Poodle, West Highland White Terrier, and Whippet. There were 14 male dogs (61%, of which eight were neutered and six were entire), and nine females (39%, of which eight were neutered and one entire). The median age at presentation was five years old (range: 1–13 years) (Supplementary Table S2).

**Figure 1:**
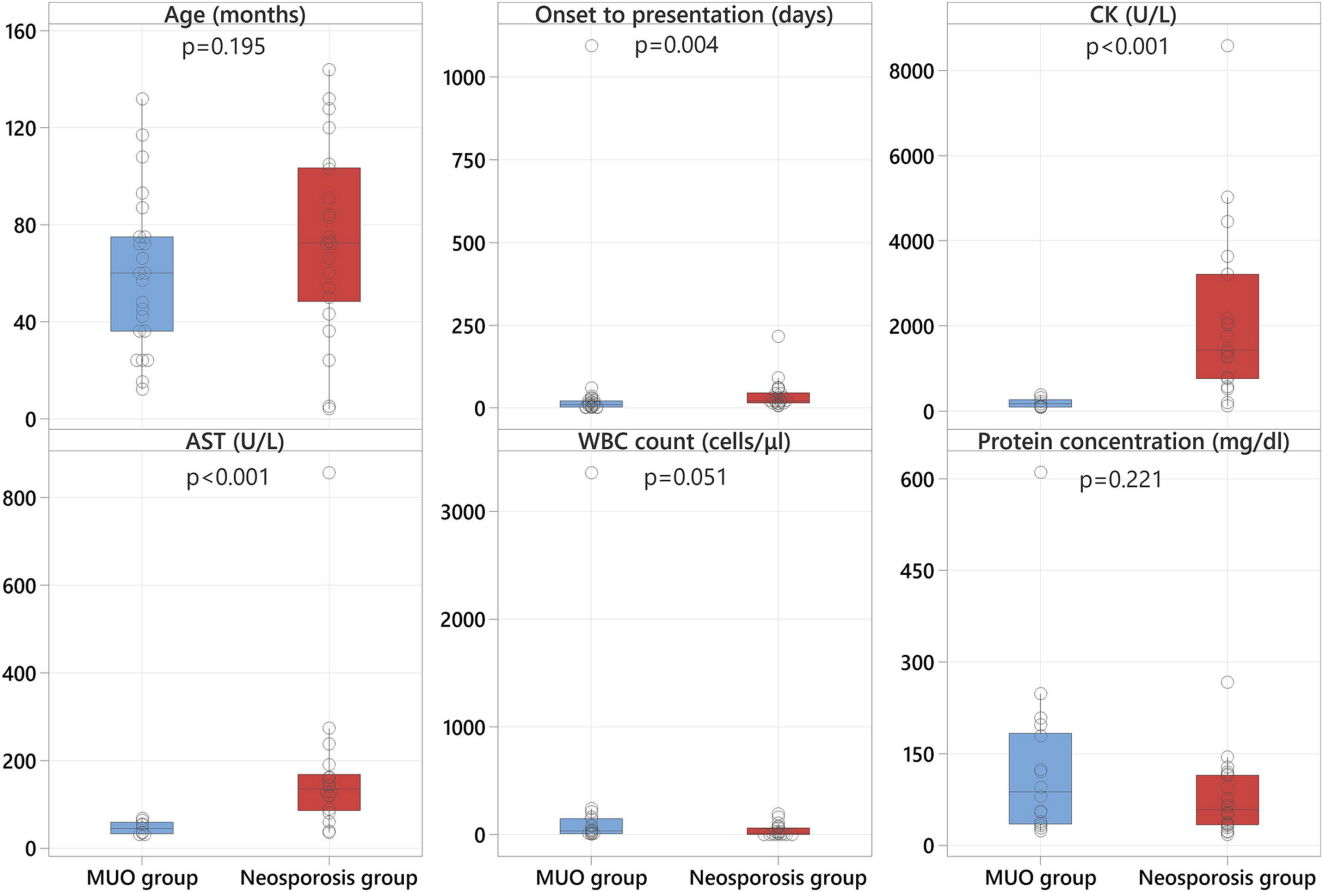
Boxplots representing the MUO group (blue shading) and the neosporosis group (red shading) revealing significantly higher CK and AST values in the neosporosis group, in addition to a significantly greater number of days between onset and referral in these cases.

No statistically significant differences in sex (*p* = 0.091) or age (*p* = 0.195) were observed between the two groups (Figure 1).

### 3.2 Neurological signs

The median time from onset of the clinical signs to presentation at the referral hospital was 21 days for the neosporosis group (range: 5–215 days) and 10 days (range: 1–1,095 days) for the MUO group (Supplementary Tables S1, S2). The time from onset of clinical signs to presentation was significantly different between the two groups (*p* = 0.004), with the neosporosis cases having a longer history of clinical signs before referral (Figure 1).

The neuroanatomic localizations identified in the neosporosis group were as follows: multifocal (15/22; 68% - of which three included a neuromuscular localization, eight included the cerebellum, and four the central vestibular system), cerebellum (5/22; 23%) and forebrain (2/22; 9%). From this group, only two animals (9%) had masticatory muscle atrophy recognized during neurological examination (Supplementary Table S1). The neuroanatomic localizations identified in the MUO group were multifocal (12/23; 52% - of which 11 included the brainstem and nine included the forebrain), forebrain (7/23; 30%), central vestibular system (2/23; 9%) and cerebellum (2/23; 9%). None of the dogs in the MUO group presented with masticatory muscle atrophy or generalized muscle atrophy (Supplementary Table S2). None of the MUO dogs had a neuromuscular localization at initial assessment compared to three of the neosporosis cases, but this difference was not statistically significant (*p* = 0.108). Four dogs in the MUO group presented with a history of epileptic seizures compared to two dogs in the neosporosis group, with an additional dog in the latter group having collapsing episodes which were not further characterized.

### 3.3 Diagnostic investigation findings

Results of serum CK and AST activity were available for all but three dogs in the neosporosis group (19/22, 86%), with an additional dog having only the CK activity assessed. In this group, the median value for CK activity was 1,423 U/L (range: 115–8,584; reference 10–200), and for AST activity was 134.5 U/L (range: 35–857; reference 0–50) (Supplementary Table S1). In the MUO group, these data were available for 9/23 dogs (39%), with a median CK activity of 161 U/L (range: 79–374) and a median AST activity of 45 U/L (range: 31–68) (Supplementary Table S2). Both the CK and AST activities were significantly higher in the neosporosis group compared to the MUO group (*p* < 0.001) (Figure 1).

All dogs had the results of either *Neospora caninum* serologic testing or PCR testing available. In the neosporosis group, all 22 dogs were assessed for serum *Neospora* antibodies titers, and 19/22 (86%) also had PCR testing. The serologic titer was ≥1:800 (range: 1:800–1:6400) in 21/22 dogs. One dog had an increased titer of 1:400 and also had a positive *Neospora* PCR. Of the 19 dogs that had PCR testing, 10 (53%) were positive (Supplementary Table S1). In the MUO group, 19/23 dogs (83%) had serologic testing for *Neospora caninum,* and four dogs (17%) had both serology and PCR performed. The results of these tests were negative in all dogs (Supplementary Table S2). There was no statistically significant difference between the neosporosis and MUO groups in regard to PCR results (*p* = 0.104). The antibody titers could not be included in the statistical analysis because the highest positive dilution was unknown in most cases.

All cases in the neosporosis group had CSF analysis performed at the time of investigations. This revealed a pleocytosis in 15 dogs (68%) with different cellular populations: a mixed pleocytosis with significant eosinophilic component in 6 dogs, a mononuclear pleocytosis in 5 dogs, and mixed pleocytosis in 4 dogs. Hemodilution was detected in 3/15 dogs with concurrent pleocytosis, and another seven dogs (32%) had normal cell counts; of these seven dogs, three had albuminocytologic dissociation. The median CSF WBC count in the neosporosis group was 10 cells/μL (range: 0–192; reference <5), and the median CSF protein concentration was 58.2 mg/dL (range: 17.6–266.8; reference <30) (Supplementary Table S1). Cerebrospinal fluid sampling was not attempted in five dogs of the MUO group (22%) because of concerns related to increased intracranial pressure. All other MUO cases (18 dogs) had a mononuclear or lymphocytic pleocytosis as per the inclusion criteria. The median CSF WBC count in the MUO group was 33 cells/μL (range: 3–3,360) and the median CSF protein concentration was 87.2 mg/dL (range: 23.4–610). None of the MUO cases had albuminocytologic dissociation but one had additional hemodilution (Supplementary Table S2). There was no statistically significant difference for the CSF WBC count (*p* = 0.051) nor for the CSF protein concentration (*p* = 0.221) between groups (Figure 1).

### 3.4 Masticatory muscle assessment on MRI

**Figure 2:**
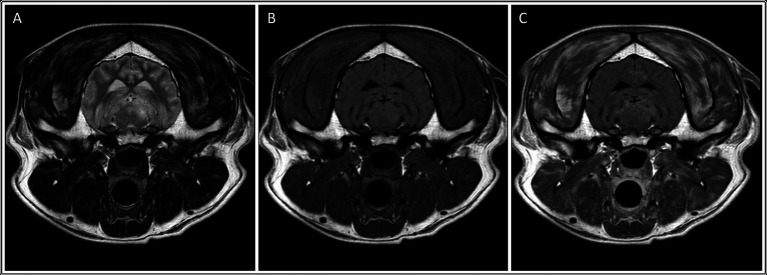
Transverse: T2-weighted **(A)**, T1-weighted **(B)**, and T1-weighted post contrast images **(C)** of a dog with muscle changes graded as severe, multifocal and bilateral (involving the temporalis, masseter, medial pterygoid and digastricus muscles), without apparent muscle atrophy. *This dog was subsequently diagnosed with neosporosis*.

**Figure 3:**
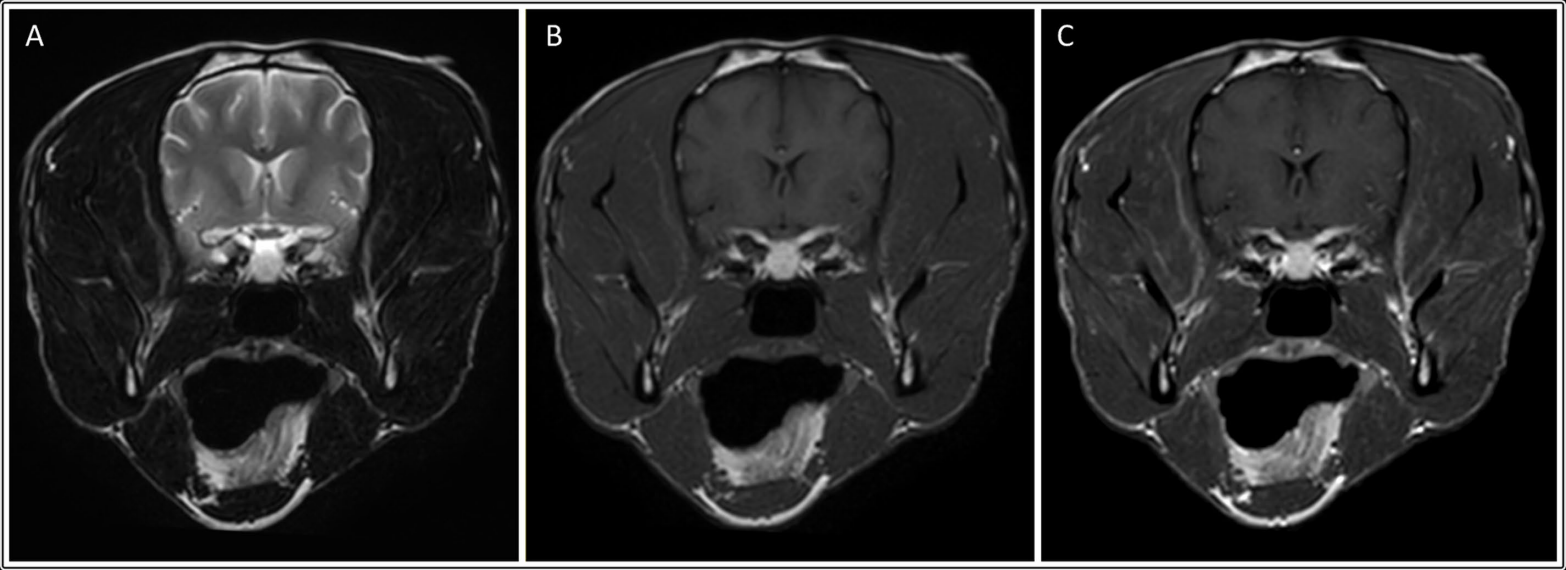
Transverse: T2-weighted **(A)**, T1-weighted **(B)**, and T1-weighted post contrast images **(C)** of a dog with muscle changes graded as mild, multifocal and bilateral (involving the temporalis, masseter and medial pterygoid muscles), in addition to mild asymmetric muscle atrophy (involving the right temporalis muscle). *This dog was subsequently diagnosed with neosporosis*.

In the neosporosis group, six dogs (27%) had masticatory muscle changes observed on MRI. The involved breeds were Labrador Retriever (*n* = 2) and one of each of the following breeds: Lurcher, Cocker Spaniel, French Bulldog and Boxer (Supplementary Table S1). The changes in both Labradors and the French Bulldog were graded as severe (grade 2) (Figure 2), whereas the changes in the other three dogs were graded as mild (grade 1) (Figure 3). Of these six dogs, two of those graded mild and one graded severe were also considered to have mild masticatory muscle atrophy. The atrophy was asymmetric in one of these dogs (severity grade 1) and symmetric in the other two cases. There was one Greyhound with no visible masticatory muscle lesions but that was considered to have a mild, symmetric muscular atrophy. Age of the dogs did not differ significantly between those with and without muscle atrophy (*p* = 0.448). Of the three dogs with the clinical evidence of neuromuscular disease (i.e., neuroanatomic localization including “masticatory muscles” or “neuromuscular system”), one had muscle changes graded 2 (Labrador Retriever), one had muscle changes graded 1 (Boxer), and one had no masticatory muscle changes (Greyhound).

In all six dogs with neosporosis and masticatory muscle changes, the muscular changes were bilateral, multifocal, and asymmetric, appearing as ill-defined striations or diffuse, patchy areas with abnormal signal intensity (Figures 2, 3). These lesions were hyperintense on T2W and T2W FLAIR images (*n* = 6), and hyperintense (*n* = 1) or isointense (*n* = 5) on T1W images. The lesions showed contrast enhancement in all dogs (*n* = 6), being marked for the three dogs with grade 2 changes, and mild in the three with grade 1 changes. In two of the dogs with mild muscular changes, the lesions were most visible in the T1W sequences post-gadolinium injection. The distribution of the lesions appeared otherwise similar in the dogs with mild changes and those with severe changes. All visible masticatory muscles (both temporal, both masseter, both pterygoid muscles, and in one dog also the digastricus muscles) were affected in all three dogs with grade 2 changes. Of the three dogs with grade 1 changes, one had only both temporal muscles affected, one had both temporal and masseter muscle lesions, and one had muscle changes in both temporal, both masseter and the left pterygoid muscles (Supplementary Table S1).

**Figure 4:**
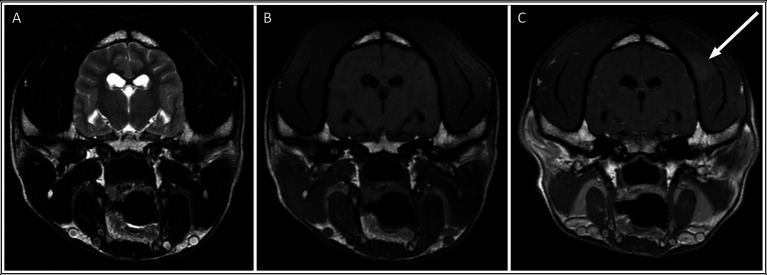
Transverse: T2-weighted **(A)**, T1-weighted **(B)**, and T1-weighted post contrast images **(C)** of a dog with muscle changes graded as mild, focal and unilateral (within the left temporalis muscle), without apparent muscle atrophy. *This dog was subsequently diagnosed with MUO*.

In the MUO group, only one dog (4%) had masticatory muscle changes. This was a Border Collie with a focal, unilateral, mild (grade 1) lesion of the left temporal muscle, adjacent to the skull (Figure 4). The lesion was relatively ill-defined and only visible on the post-contrast sequence. The masticatory muscle bulk of this dog was otherwise symmetric, and no atrophy was observed (Supplementary Table S2).

The severity of the masticatory muscle changes (*p* = 0.033), the presence of muscle atrophy (*p* = 0.049) and presence of contrast enhancement (*p* = 0.033) were significantly different between the MUO and the neosporosis groups; dogs with neosporosis were more likely to have masticatory muscle changes (*p* = 0.047) and atrophy, and these changes were more severe in the neosporosis group. The presence of muscle changes had a sensitivity of 27% (95% exact CI 11–50%) and a specificity of 96% (95% exact CI 78–100%) to predict a diagnosis of neosporosis. In this regard, the odds ratio was 8.25 (95% CI 0.90–75.40) but was not statistically significant from 1 (*p* = 0.062). No difference between groups was found in regard to the presence of focal/multifocal changes (*p* = 0.143), unilateral/bilateral changes (*p* = 0.143) and symmetry of the muscle bulk (*p* = 0.490).

**Figure 5:**
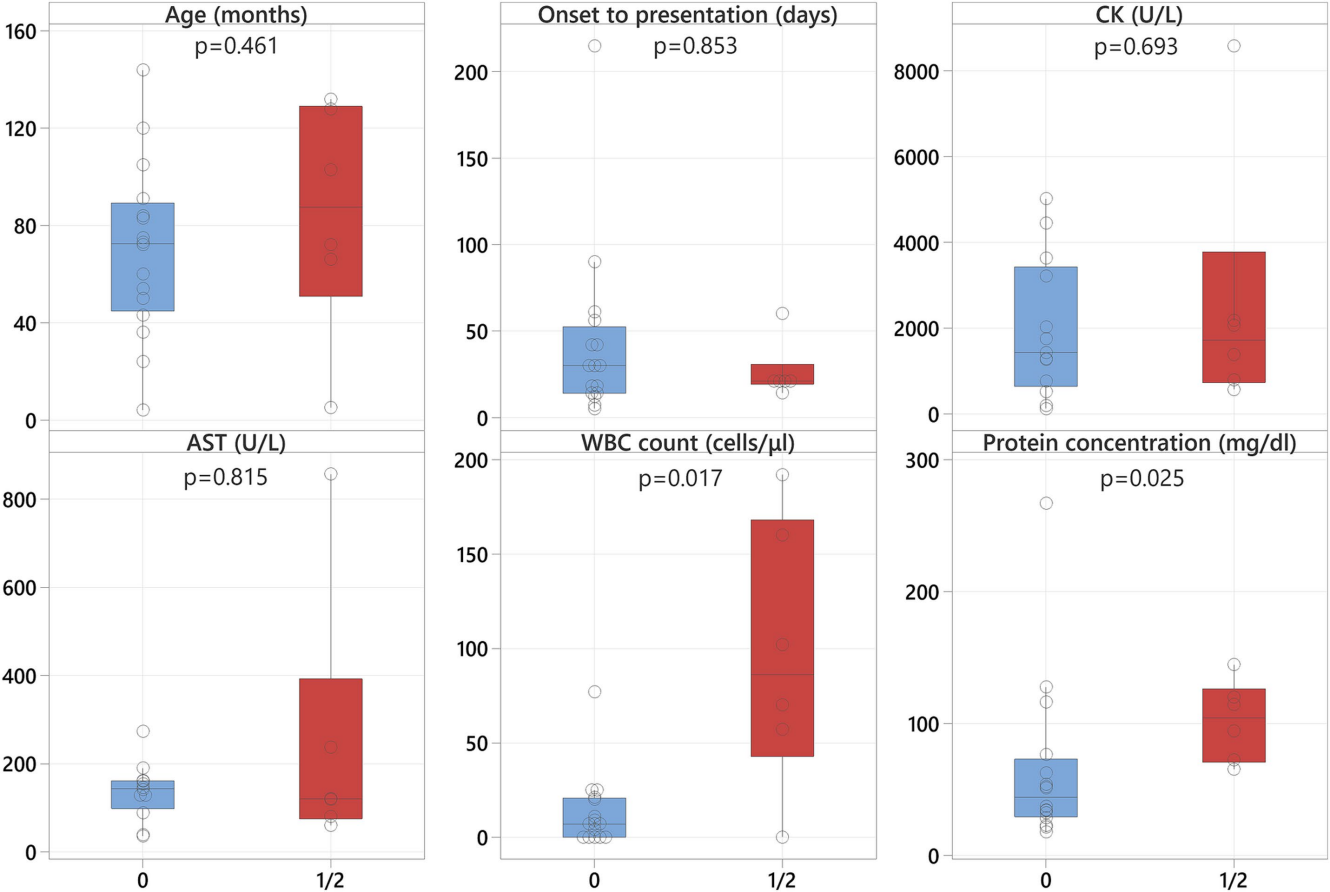
Boxplots representing the neosporosis dogs without muscle changes (blue shading) and those with muscle changes (red shading) revealing significantly higher CSF WBC counts and protein concentrations in the latter.

Within the neosporosis group, dogs with masticatory muscle changes (grades 1 and 2 grouped together) had significantly higher CSF WBC counts (*p* = 0.017) and CSF protein concentrations (*p* = 0.025) compared to dogs with no visible masticatory muscle lesions (grade 0). The median values for the CSF WBC count were 86 cells/μL (range: 0–192) for dogs with masticatory muscle changes compared to 7 cells/μL (range: 0–77) in those without (Figure 5). The median values for the CSF protein concentrations were 104.3 mg/dL (range: 65.2–144.6) for dogs with masticatory muscle changes compared to 44 mg/dL (range: 17.6–266.8) in those without (Figure 5). In addition, all dogs with masticatory muscle lesions (*n* = 6) had a positive *Neospora* PCR, compared to only four of the remaining 13 dogs with PCR testing and no visible muscle changes (*p* = 0.011). No statistically significant differences were identified regarding age, time from onset of clinical signs to presentation, and CK and AST activities between the neosporosis dogs with masticatory muscle changes and those without (Figure 5).

The trigeminal nerve was normal on MRI for all dogs in both groups. None of the dogs with masticatory muscle changes had seizures or any other reported abnormal or collapsing episodes in their clinical history, including the single dog in the MUO group.

## 4 Discussion

The present study assessed the presence of MRI changes in the masticatory muscles of dogs with neosporosis in comparison to dogs diagnosed with MUO. In light of the potential for overlap between the intraparenchymal brain changes seen on MRI for these two conditions and the delay in receiving the results of serum or CSF infectious disease testing, markers for the presence of infectious disease at the time of scanning would allow prompt initiation of the most appropriate treatment. Overall, 27% of the dogs (6/22) in the neosporosis group had masticatory muscle changes, which were bilateral and multifocal in all cases. Half of these changes were categorized as mild and the remainder as severe. Only one dog (4%) from the MUO group had a temporal muscle lesion that was focal and unilateral. While it was visible on both the T1W SE post contrast and 3D T1W post contrast images, the focal lesion in the single MUO case was only visible on post-gadolinium sequences, with a normal masticatory muscle appearance on T2W, T2W FLAIR and T1W images. It is therefore suspected that this may have represented an artifact due to the coil (intensity non-uniformity) rather than a true pathologic lesion associated with the MUO. There was also no history of seizures or falls in this dog to suggest that the focal lesion may have resulted from external trauma. Statistical analysis revealed that, in this population of cases, dogs with neosporosis were not only more likely to have masticatory muscle changes than dogs with MUO (*p* = 0.047), but also that within this group the muscle lesions were more severe (*p* = 0.033).

*Neospora caninum* is an obligate, intracellular protozoan parasite with a canine definitive host and a predominately bovine intermediate host (27). It can affect both the central and peripheral nervous system of dogs causing many different clinical pictures, including polyradiculoneuritis in puppies, as well as meningoencephalomyelitis and necrotising cerebellitis in adult dogs (8, 22, 28). Moreover, *Neospora* can also invade the skeletal muscles and cause a generalized polymyositis (14, 29). Although less common, *Neospora* has been also reported to infect other body systems as a result of extensive tachyzoite dissemination, causing conditions including myocarditis, fibrinohaemorrhagic enteritis, pneumonia, peritonitis and dermatitis (14). Considering the predilection of this parasite for striated muscles in the canine host and the previously reported increase in muscle enzymes observed in dogs with *Neospora* meningoencephalitis compared to those with non-infectious (suspected immune-mediated) meningoencephalitis (25), it would appear logical that these patients may have concurrent muscular changes visible on MRI. However, this has not been assessed in the veterinary literature to date. The most common presentation for neosporosis in adult dogs is intracranial disease, with MUO being the most important differential diagnosis prior to starting treatment. The masticatory muscles, rather than other axial or appendicular muscles, were therefore chosen as the muscles of interest for this study as they are visible on routine brain MRI sequences used for the majority of *Neospora* and MUO cases alike, meaning that no additional scans or tests would be needed in such cases.

All six dogs with *Neospora* meningoencephalitis and masticatory muscle lesions on MRI had multifocal and bilateral changes, whereas the lesion observed in the single MUO case was focal and unilateral. However, statistical analysis for the presence of focal or multifocal, as well as bilateral or unilateral, muscle changes did not reveal statistically significant differences between the two groups, possibly due to the small sample size. However, dogs in the neosporosis group were more likely to have muscular atrophy and contrast enhancement of the muscular changes than dogs in the MUO group (*p* < 0.05). More specifically, in the neosporosis group, the presence of masticatory muscle abnormalities was associated with masticatory muscle atrophy in 3/6 dogs, and one additional neosporosis dog had mild, symmetric muscular atrophy in the absence of visible changes in masticatory muscle signal intensity. This underlies how the presence of masticatory muscle signal intensity changes does not necessarily correlate with muscular atrophy. This finding may, in part, be related to disease stage, as acute inflammatory myopathies can result in muscle swelling whereas only in the more chronic stages of the disease does muscular atrophy occur (30–32). Other factors that could result in masticatory muscle atrophy were also considered in this study, including animal age and administration of corticosteroids prior to investigations. It may be hypothesized that aging could be associated with muscular atrophy as a natural process of sarcopaenia (33). However, the age at presentation of the dogs classified as having masticatory muscular atrophy on MRI varied widely (36–128 months) and there was no statistical association between the presence of masticatory muscle atrophy and age for the dogs in this study (*p* = 0.448). There was also no clinical history of trigeminal nerve disease in any of the dogs in this study and the trigeminal nerves were rated as normal on MRI in all dogs. Therefore, the presence of denervation atrophy secondary to trigeminal nerve (motor component) disease was considered highly unlikely. This was an important exclusion criterion since trigeminal neuropathy can affect the masticatory muscle bulk and appearance on MRI by causing atrophy and increased signal intensity on T1W images, as well as contrast enhancement of the atrophied muscles (34). Regarding the administration of corticosteroids, none of the dogs in the MUO group received corticosteroids prior to investigation. Of the 22 dogs with *Neospora caninum*, 17 did not receive corticosteroids prior to presentation, this information was not available in two dogs and the remaining three dogs had received prednisolone prior to investigations. While the administration of prednisolone may have contributed to the mild masticatory muscle atrophy observed in two of these dogs (dogs 7 and 15 in Supplementary Table S1), the concurrent presence of distinctive, multifocal, bilateral, contrast-enhancing intramuscular changes in these two dogs cannot be explained by the administration of corticosteroids alone, and an infectious masticatory myositis remains the most likely diagnosis in these cases.

Depending on the coil used for image acquisition and on the recumbency of the dog at the time of imaging (dorsal versus sternal), the masticatory muscles frequently appeared mildly asymmetric. However, the method chosen to review the MRI studies (consensus of three reviewers) aimed at reducing the risk of incorrectly classifying the masticatory muscles as asymmetric or atrophied related solely to positioning or coil type. This situation mimicked that at the authors’ establishment, where radiologists and neurologists frequently discuss cases and assess scans in real time, providing the opportunity to discuss each case and be as objective as possible in the characterization of the masticatory muscle changes. In addition, with the aim of further reducing subjectivity during data collection, the severity of masticatory muscle changes, degree of masticatory muscle atrophy and degree of contrast enhancement were categorized as either absent, mild or severe, with “moderate” not being used. Volumetric measurements of the individual masticatory muscles in each case could be used to more objectively determine masticatory muscle bulk. However, without a baseline measurement prior to the onset of MUO or neosporosis, this would not have allowed the determination of relative muscle atrophy for each individual case and was therefore not performed in this study. In addition, the wide natural variation in masticatory muscle bulk between dogs of different breeds means that a single expected volumetric value could not be used to determine the presence of atrophy. This was also the reason why the reviewers were blinded to all case information other than breed at the time of assessment.

The MRI features of inflammatory myopathies depend on the stage of the disease. In acute cases, the muscles appear hyperintense in T2W images due to the development of oedema and accumulation of inflammatory infiltrates in the affected muscles, which cause a prolongation of the T2 relaxation times (35–37). T1 relaxation times are less affected by the presence of oedema and, therefore, the abnormal muscles generally appear T1W isointense or slightly hypointense (35, 36, 38, 39). In chronic myositis, the muscle lesions can appear hyperintense in both T1W and T2W images due to fat infiltration, associated with muscle atrophy. However, in the presence of fibrosis, the affected muscles become isointense to hypointense on T1W and T2W images (36, 38, 40). In this study, the signal intensities of the muscle lesions in all but one of the six neosporosis dogs reflected the presence of acute myositis. In the remaining case, a Labrador Retriever with a two-week history of clinical signs, the masticatory muscle changes were hyperintense on T1W images, did not suppress in the short tau inversion recovery (STIR) sequence, and did not show signal void in the T2*-GRE (gradient echo) weighted sequence. This would suggest that these lesions were unlikely to represent early fat infiltration (41) or intramuscular hemorrhage, respectively. The other differential diagnoses for a T1W hyperintense signal are the presence of melanin, proteinaceous substances and mineralization (most commonly microcalcifications, but manganese, copper or iron can also appear hyperintense on T1W images) (42). In the absence of a muscle biopsy, it is not possible to determine the underlying cause of the unusual signal characteristics in this case. However, the authors hypothesis that the most likely source for the T1W hyperintensities was the presence of proteinaceous material and/or inflammatory cells, with intramuscular hemorrhage considered unlikely. Indeed, in humans with necrotising myopathies, muscle lesions can be partially T1W hyperintense due to the presence of methaemoglobin, proteinaceous material or, in the chronic phase of the disease, fat (35, 43).

Several previous reports of dogs with immune-mediated masticatory myositis (MM) described similar MRI findings to those observed in the dogs with *Neospora caninum* in this study (30, 31, 44). Equally, histopathologic specimens of muscle affected by MM or *Neospora* myositis can appear very similar if the parasite is not directly identified, which can be a frequent occurrence. Indeed, eosinophilic muscular infiltration was commonly found both in dogs with MM and with infectious myositis (29) and, in the same study, only 1/28 dogs with generalized protozoal myopathy had *Neospora caninum* cysts detected in a muscle biopsy. Overall, this underlines how immune mediated and infectious masticatory myositis may have a similar MRI and histopathologic appearance. However, given that so far, no overlapping syndrome with concurrent MUO and immune mediated myositis has been reported in dogs, and in light of the results of the statistical analysis in this study, it can be postulated that the simultaneous presence of MRI features consistent with myositis and meningoencephalitis is more likely consistent with infectious disease and, in particular, with protozoal infection.

The prevalence of epileptic seizures in this study population was assessed given that, in the authors’ experience, focal or multifocal areas of T2W hyperintensity or, more rarely, diffuse changes can sometimes be observed in the masticatory muscles postictally. These abnormalities are likely due to intrinsic trauma (i.e., banging of the head against the floor or furniture), but bilateral symmetric T2W hyperintensities around the rami of the mandibles have also been observed by the authors. Given that none of the dogs with masticatory muscles changes had seizures in their clinical history, and that all dogs which were reported to have had seizures did not have masticatory muscles changes, this may be less relevant than hypothesized. However, given the small size of the study population, it cannot be excluded that seizures may occasionally result in masticatory muscle abnormalities that could mimic those seen with infectious or non-infectious pathologies.

As previously reported by Fisher et al. (8), Retriever breeds (i.e., Labrador and Golden Retriever), as well as Greyhounds and their crossbreeds (Whippet and Lurcher) were overrepresented in the neosporosis group in this study, with a prevalence of 18, and 32%, respectively. Moreover, two of the six dogs with masticatory muscle abnormalities were Labrador Retrievers and they both had severe changes. This finding, in association with the previous data suggestive of a predisposition for neosporosis in this breed (45, 46), may suggest that Labradors are also susceptible to develop a more severe form of the disease, or that the parasite has a greater tendency to invade the striated muscles in this breed. However, there is currently no scientific evidence to explain this assumption and the high prevalence of this infectious disease in Retrievers may simply reflect increased exposure secondary to the working nature of the breed, associated exposure to rural environments, and the anecdotal tendency of Retrievers, in particular Labradors, to polyphagia or pica with infection occurring by ingestion of intermediate hosts containing the organism. Interestingly, dogs with neosporosis had a longer clinical history before referral compared to the MUO cases (*p* = 0.004), which was suggestive of a more chronic and subtle progression of the clinical signs with neosporosis.

Regarding the clinical pathology results, as previously observed in a recent study (25), the CK and AST activities were also significantly higher in the neosporosis group. This finding is not unexpected given the predilection for skeletal muscles by *Neospora*, and a mechanism for MUO alone to increase serum muscle enzyme levels has not yet been reported. However, CK and AST levels were available in only 39% of MUO cases compared to 86% of *Neospora* cases in this study. Although CSF WBC count and CSF protein concentration subjectively appeared to be higher in the MUO group, these data did not achieve statistical significance, potentially due to the small size of the study groups.

When dogs with muscle changes and those with normal masticatory muscle signal intensity were compared within the neosporosis group, these two groups did not differ with respect to age and time from onset of clinical signs to presentation. However, dogs with *Neospora* and masticatory muscle lesions had significantly higher CSF WBC counts, higher CSF protein concentrations and were more likely to have a positive CSF PCR for *Neospora caninum* compared to those with *Neospora* but without visible masticatory muscle lesions. These findings may reflect a greater severity of the infection in dogs developing muscle lesions visible on MRI but correlation with histopathology would be required to further investigate this hypothesis. Interestingly, the CK and AST activities were not significantly different between these two groups to suggest more severe muscle damage in the cases with visible masticatory muscle lesions. However, in the absence of MRI of other body regions, the presence of distant muscle lesions in cases with higher muscle enzymes cannot be excluded.

The limitations of this study include the small study population which will have affected the power of the statistical analysis, and its retrospective nature which resulted in a lack of uniformity of the MRI parameters and protocols. This was also, in part, related to the origin of the MRI studies which derived from two referral hospitals over an eight-year period. Moreover, despite the observers being blinded to the diagnosis of the screened animals and being asked only to evaluate the muscles for any changes, while trying to disregard the brain appearance, the presence of brain MRI changes that may have been considered more suggestive for one or the other disease, may have inadvertently biased their analysis. In this study, the focus was set on only the appearance of the masticatory muscles as a possible factor which could help to differentiate between MUO and neosporosis and, therefore, the observers were required to concentrate on changes affecting these structures only. Given the known breed predispositions for neosporosis and MUO respectively, the authors may have also been biased during assessment by not being blinded to the dogs’ breeds. However, this was considered important information to aid assess for the presence or absence of muscle atrophy, since there is a wide natural variation of normal masticatory muscle bulk between different canine breeds. Another limitation of this study was the absence of CSF results for five of the MUO cases. However, this situation is not uncommon in clinical practice and CSF changes are frequently non-specific, with considerable overlap between changes seen in neosporosis and MUO cases. In addition, all of these cases had a negative *Neospora* serology and/or PCR, and the subsequent follow-up and response to immunosuppressive treatment supported the clinical diagnosis of MUO. Similarly, the lack of brain and/or muscle biopsy examination through histopathology, PCR testing, immunohistochemistry and/or *in situ* hybridization in all cases, both to confirm MUO and to exclude neosporosis, means that it cannot be completely excluded that some of the MUO cases used for this study had an alternative diagnosis, or were even affected by neosporosis. This study only included dogs with a final diagnosis of MUO and *Neospora caninum*. Therefore, while the presence of masticatory muscle lesions had a specificity of 96% for the diagnosis of *Neospora caninum* in this study, it cannot be excluded that other protozoal (e.g., *Toxoplasma* gondii), or infectious causes in general, may also result in similar masticatory muscle lesions to those observed in the dogs with neosporosis.

In conclusion, although diagnostic confirmation for canine inflammatory meningoencephalitis in dogs should be obtained by histopathologic examination of a brain biopsy, given the high costs of this procedure and the associated potential for morbidity, this is currently infrequently performed and clinicians have to rely on the combination of clinical pathology results, MRI abnormalities and treatment response to guide clinical decisions. In this study, characteristic multifocal, bilateral, consistently contrast-enhancing masticatory muscle lesions, with occasional concurrent muscle atrophy, were observed in more than one quarter of dogs with neosporosis, whereas similar changes were not observed in any of the MUO patients. Hence, when combined with other clinical information, such as the presenting history, muscle enzyme activity and the appearance of any intraparenchymal brain lesions, such muscular changes should be considered an important additional finding that could aid in distinguishing between MUO and *Neospora caninum* at the time of imaging, guiding prompt initiation of antibiotic treatment. However, this study also demonstrated that the absence of masticatory muscle changes in dogs with imaging features consistent with meningoencephalitis does not exclude a possible infectious origin, with a sensitivity of only 27%. In these ambiguous cases, serological testing and/or PCR or muscle biopsy are still required to exclude protozoal infection (47). However, in light of current recommendations regarding responsible use of antimicrobials, the authors do not feel that these results support the widespread use of antimicrobials in all such cases pending these results. It could also be suggested that, in an ideal world, starting immunosuppressive treatment should be delayed until these results are available. However, particularly in animals showing a rapid clinical deterioration, clinicians should still act on case-to-case basis and consider all aspects of the case; if MUO is strongly suspected based on signalment (i.e., toy or small breeds), history (i.e., short time from onset of clinical signs to presentation), clinical pathology results (i.e., unremarkable CK and AST activities, mononuclear CSF pleocytosis) and imaging findings, starting an anti-inflammatory or even immunosuppressive dose of corticosteroids may be appropriate at the time of imaging. Similarly, if neosporosis is still strongly suspected despite the absence of masticatory muscle changes on MRI, in light of the signalment (i.e., young Labrador Retriever or Sighthound), clinical pathology results (i.e., increased CK and AST activities, eosinophilic CSF pleocytosis) and/or MRI findings (i.e., necrotizing cerebellitis) (8), starting early antimicrobial treatment targeting *Neospora caninum* should be considered.

## Data Availability

The original contributions presented in the study are included in the article/Supplementary material, further inquiries can be directed to the corresponding author.
